# The use of the pediatric physeal slide-traction plate in the treatment of neer–horwitz grade IV proximal humeral fractures in children: A case report and literature review

**DOI:** 10.3389/fsurg.2022.960541

**Published:** 2022-09-14

**Authors:** Le Qi, Yikun Jiang, Yanbing Wang, Chuangang Peng, Dankai Wu

**Affiliations:** Department of Orthopedics, The Second Hospital of Jilin University, Changchun, China

**Keywords:** pediatric physeal slide-traction plate, proximal humeral fracture, children, case report, literature review

## Abstract

**Background:**

Proximal humeral fractures (PHFs) are rare in children. Currently, the recommended surgical methods for severely displaced PHFs are closed reduction and percutaneous fixation using K-wires or intramedullary nailing, which can't provide firm internal fixation, especially for older and high-weight children. This study aimed to introduce a novel surgical approach, pediatric physeal slide-traction plate fixation (PPSP), for Neer–Horwitz grade IV PHFs in children.

**Case summary:**

A 9-year-old boy presented with left shoulder pain and swelling due to a car accident. Physical examination revealed a positive shoulder deformity and local tenderness. On physical examination, we palpated bone friction without vascular and nerve damage. Based on imaging findings, we diagnosed Neer-Horwitz grade IV PHF. In view of the patient's condition, we performed PPSP after careful communication with the patient's parents. After 22 months of follow-up, the patient's left shoulder function was satisfactory, and there was no restriction of activities.

**Conclusion:**

According to previous studies, PPSP is only used for femur fractures. To the best of our knowledge, this is the first in the treatment for PHFs. Given the satisfactory outcomes, it is a safe and effective method and may provide a reference to cure analogous patients in the future.

## Introduction

Proximal humeral fractures (PHFs) are relatively rare, accounting for approximately 3% of all pediatric fractures (1). It is mainly caused by falls, sports, car accidents, or birth traumas, and the incidence of sports-related injuries increases with age (2, 3). The major mechanism of PHFs is that when falling backward, the arm lands, and the upper limb adducts with elbow extension. Simultaneously, the shoulder extends and rotates outward. In PHF, metaphyseal fractures account for about 70% of cases and epiphyseal separation for the remaining 30% (1, 4, 5).

The epiphyseal plate (also known as epiphyseal cartilage, growth plate, or epiphyseal growth plate) is particularly important during the growth and development of children. It is more susceptible to damage than tendons, ligaments, and joint capsules and its injury is more common in long bones. The epiphyseal plate is the growth and development site of bone during the growth period and functions in longitudinal and transverse growth. Only normal cell function, orderly band arrangement, and complete blood supply are important for maintaining the physiological function of the epiphyseal plate. The epiphysis of the proximal humerus is responsible for 80% of humeral length growth (6). When PHF occurs, the epiphysis is directly damaged, resulting in loss of blood supply to both ends and dysfunction of the epiphyseal plate (7). This is followed by early epiphyseal and bone bridge formation. Patients may have residual bilateral limb length differences, progressive angular malformation, and limb dysfunction (8, 9).Therefore, caution should be exercised when treating PHFs in children.

There is a great potential for bone healing and spontaneous remodelling after PHF in children. Some people believe that patients aged >12 years with obvious displacement need surgical treatment (10), whereas others believe that only patients aged <8 years can completely remodel for Neer–Horwitz grade IV fracture. The residual deformity of patients greatly increases with age, and surgical treatment should be performed for all patients (11). Therefore, it is difficult to assess the effect of age on clinical outcomes (12). However, it is currently agreed that obvious displacement after fracture or serious vascular or nerve damage require surgical explorationsurgical treatment, including percutaneous Kirschner wire and flexible plate and screw fixation (4, 13).

In previous clinical and animal studies, pediatric physeal slide-traction plate fixation (PPSP) was only used in the lower limb fractures and not in upper limb fractures. Therefore, we proposed this procedure for Neer–Horwitz grade IV PHF in children. In this case, PPSP can be prolonged with proximal humeral growth, which provides reliable internal fixation without affecting epiphyseal growth.

## Case presentation

### Chief complaints and physical examinations

A 9-year-old boy experienced painful swelling and limited movement of his left shoulder after a car accident. On physical examination, we palpated bone friction without vascular and nerve damage. There was no associated neurovascular injury.

### Imaging examinations and final diagnosis

X-ray films showed discontinuity of the left proximal humerus cortical bone, belonging to the type of complete dislocation(Figure 2A). Based on the patient's medical history and examination results, we diagnosed him with Neer–Horwitz grade IV PHF on the left side.

## Treatment

### Construction of plates

**Figure 1:**
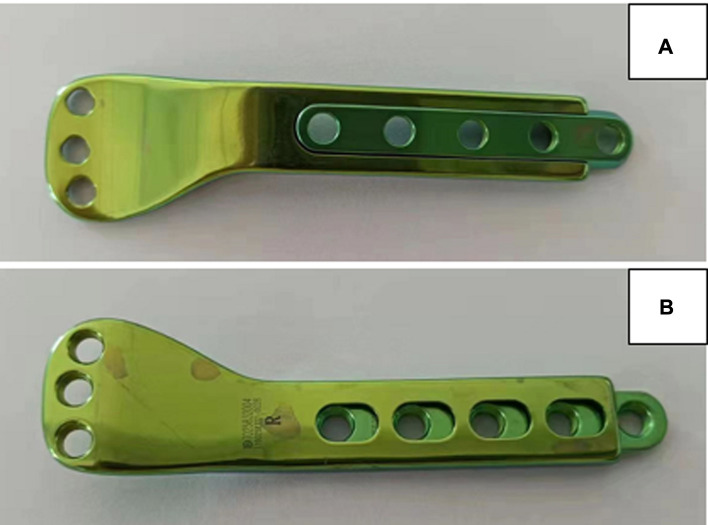
Images of the pediatric physeal slide-traction plate.

We used a 3.5-mm pediatric sliding locking plate from Double Medical Company, which is made of titanium alloy and consists of a barrel and sliding. The head and body of the plate are used to fix the epiphysis and metaphysis/diaphysis of the distal femur respectively, bypassing the growth plate. The plate was used following the same principles for fixation of the proximal humerual fracture in our case. The head of the plate is 80 mm long, 13 mm wide, and 4 mm thick. The body of the plate is 60–120 mm long, 9.3 mm wide, and 2.6 mm thick. The hole spacing is 10.0 mm. The outer diameter of the screw is 3.5 mm. The plate head has the shape of a drawer, which allows for slide traction of the body part (Figure 1).

### Surgical technique and postoperative care

**Figure 2:**
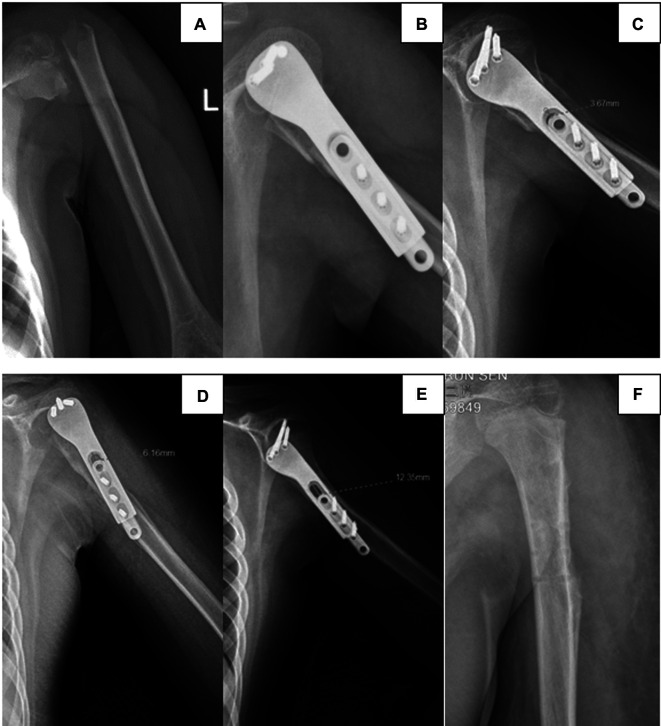
Radiographs of a proximal fracture of the left humerus in a 9-year-old boy by a traffic accident. (**A**) Preoperative lateral radiography shows proximal humeral fractures, Neer–Horwitz grade IV. (**B**) Intraoperatively, the internal fixation device is found to be in good position. (**C**) Lateral radiography at 6 weeks after surgery. (**D**) Lateral radiography at 3 months after surgery. (**E**) Lateral radiography at 6 months after surgery. (**F**) Postoperatively, slide plate removal.

A deltopectoral approach to the proximal humerus was utilised and the fracture was exposed. A proximal humeral sliding plate was placed after the fracture was reduced. We screwed one screw each at the proximal and distal ends of the PPSP to provide temporary fixation. Fluoroscopy showed that the plate screw and fracture end were in a good position, and the remaining screws were inserted (Figure 2B). Fixation works if the head and body of the PPSP slide relative to each other. The total operative time was 80 min.

No splint or abduction brace was used after surgery, and active shoulder and elbow exercises were started on the second postoperative day. The sliding length of the PPSP was measured using anteroposterior and lateral radiographs. At the last follow-up, three-dimensional computed tomography of the affected limb was performed to measure humeral length. In addition, we recorded any complications associated with the surgery.

### Outcome and follow-up

**Figure 3:**
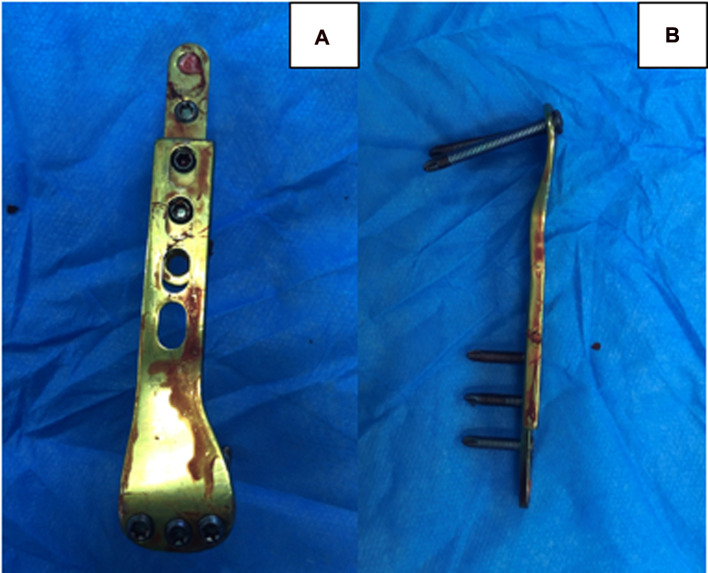
The internal fixation device is removed completely after surgery without fracture. (**A**) The front view. (**B**) The side view.

**Figure 4:**
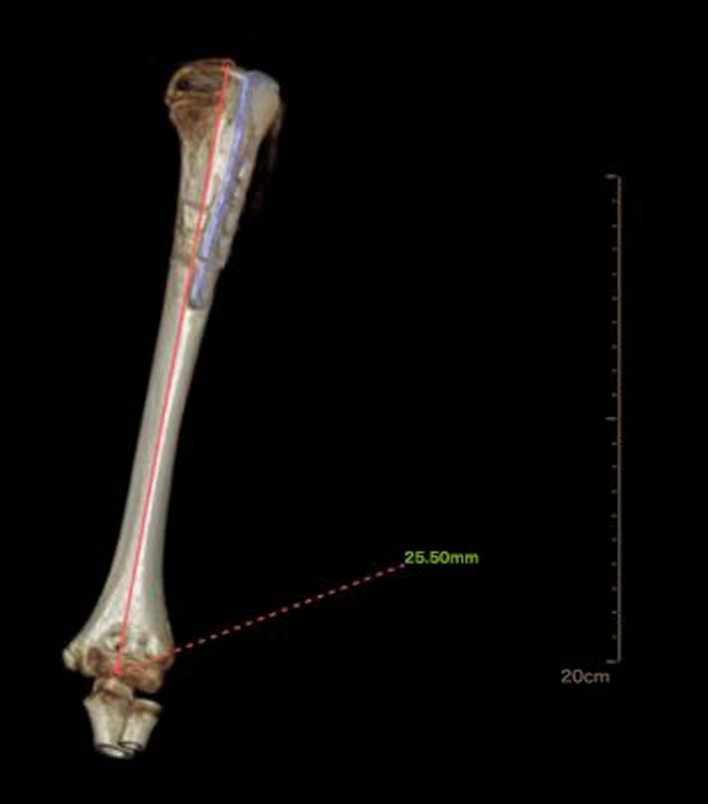
Measurement results of the length of the affected limb.

**Figure 5:**
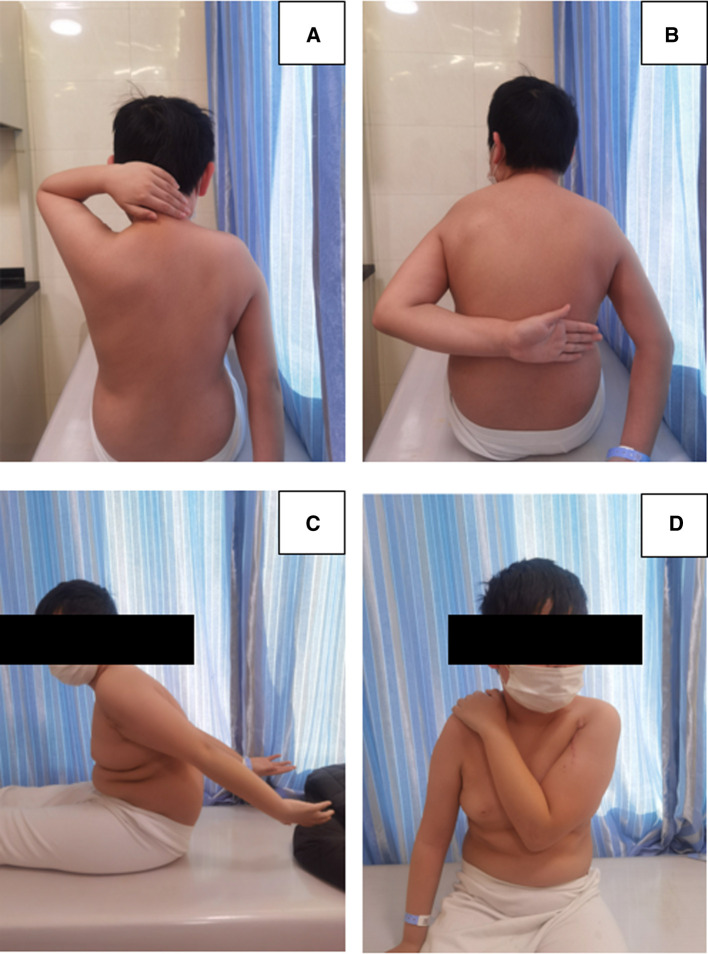
The patient's left shoulder has a normal range of motion without dysfunction after 6 months of removing the internal fixation device.

The total postoperative hospital stay was 5 days. Radiography was performed at 6 weeks and 3 and 6 months after surgery to evaluate the sliding distance of the sliding plate, which were 3.67, 6.16, and 12.35 mm respectively (Figures 2C–E). At 6 months of follow-up, radiography showed complete union of the fracture at the fracture end. The metaphyseal morphology of the affected limb was normal. The sliding plate was in a good position, and the sliding distance increased significantly. At 6 months postoperatively, the internal fixation device was removed according to the patient's recovery (Figure 2F). Intraoperatively, the internal fixation devices were completely removed without fracture or other complications (Figure 3). Then, we obtained three-dimensional computed tomography images of the affected limb to measure the length. The results showed that the length between the most proximal point of the humeral head and the most distal point of the lateral trochlear crest in the standard anatomical position was 255 mm, consistent with the normal length in children of the same age (14) (Figure 4). No upper limb discomfort or dysfunction occurred after surgery, and the range of motion of the shoulder joint was completely symmetrical (Figure 5). The Disability of Arm Shoulder and Hand (DASH) score for the left shoulder decreased from 86.67 to 17.5.

## Discussion

In children with displaced and unstable PHF, the optimal outcome is to maintain normal limb function and limb force line to the fracture end. Meanwhile, the growth and development of the epiphyseal plate don't be affected so that to ensure its normal transverse and longitudinal growth. Therefore, effective internal fixation and postoperative avoidance of secondary epiphyseal injuries are important factors.

Percutaneous Kirschner wire internal fixation is a common clinical treatment with less intraoperative trauma, shorter operative time, and reasonable cost and is favored by many clinicians. However, it is prone to the risk of humeral head perforation (15). The flexible intramedullary nail (FIN) fixation technique is also in line with the principle of minimally invasive technology, which is stable and safe enough for the surrounding soft tissues (4, 16, 17). Good outcomes, including fast healig and reliable fixtion, pain relief, and return to normal function, can be obtained with the use of FIN (18). However, the most commonly reported complication of FIN in pediatric humeral fractures is implant prominence (19). There is no consensus in the literature regarding whether or not these implants must be removed, and the optimal timing to remove it remains unknown (18). Although the traditional plate internal fixation technique provides good stability, torsion resistance, and reliable fixation of the fracture site, the plate cannot be removed early. Biological stresses in the opposite direction of epiphyseal growth can occur at later stages, severely limiting epiphyseal growth, which is a defect that can be fatal for children with PHF (20, 21).

Consequently, the conventional treatment of pediatric PHF may result in unreliable fixation and secondary epiphyseal plate injury, such as epiphyseal penetration injury, longitudinal growth inhibition, and other complications. The best surgery should depend on the patient's remodeling potential, amount of deformity, and functional needs. In view of the patient's grade IV, large angular malformation, and failure of closed reduction, open reduction was adopted. We designed a PPSP that slides along with the growth of the epiphyseal plate while providing reliable fixation for the fracture.

The PPSP system was first proposed by Lin et al. (21). In previous studies, it was applied to lower limb fractures (21–24). This set of instruments was used for the first time to treat PHFs. The head of the plate was used to fix the proximal fracture, and the body was used to fix the distal fracture. Vertical compression, three-point bending, and torsion resistance are the main indicators that are commonly used to evaluate the reliability of internal fixation. The head of the PPSP has a drawer-like groove along which the body can slide. This biomechanical design provides sufficient strength for compression, torsion, and bending (21). In clinical practice, it can ensure that the fixed limbs can resist a certain vertical pressure, torsional force, and lateral bending stress without breaking and provide a certain mechanical stability.

Greco et al. (25)have proved with animal experiments that if the pressure of internal fixation on the fracture end continues and gradually increases, it would lead to permanent dysfunction of the epiphyseal plate function when it exceeds a certain limit, and the most common consequence is the unequal length deformity of bilateral limbs left after healing. The PPSP system we used provides patients with a biomechanical environment with stable longitudinal motion. The sliding plate can slide along with the growth of the epiphysis; therefore, it does not exert pressure on the longitudinal axis of the bone, limiting the growth of the bone, and it does not cause long-term inhibition of growth or even early closure of the epiphysis plate, which was confirmed through a series of *in vitro* experiments by Lian et al. (23). In contrast, when the muscle is stretched or contracted, PPSP will slide between the head and body; therefore, the fracture end is constantly stimulated by stress. Such stimulation can promote fracture healing through hematoma absorption, osteocyte differentiation, vascular proliferation, and callus formation. Thus, the recovery time of the fracture can be shortened to some extent, and the internal fixation device can be removed as soon as possible (23).

During follow-up, we observed limited shoulder abduction dysfunction prior to removal of the internal fixation device. This is because we used an internal fixation device designed for the femur, and the head of the plate was higher than the greater tuberosity of the humerus, which limits the abduction of the shoulder joint. However, the symptoms disappeared completely after removal.

Despite the positive clinical results reported in this case report, there are some limitations, including (1) this is just a case report and nededs more cases to confirm efficacy, (2) short follow-up period, (3) lack of follow-up of suppressed epiphysis, and (4) use of a single surgeon and the lack of a multicenter study, (5) this is an off-label use of this plate which is designed for the distal femur and we need to apply for a patent in this regard. In conclusion, we should further establish a multicenter trial with a larger sample size and long-term follow-up.

## Conclusion

Here, we report a new method for treating PHF using PPSP. After 22 months of follow-up, the patient's shoulder range of motion was normal, and there was no residual limb deformity. Imaging results show that PPSP can be effectively prolonged with the growth of the epiphyseal plate. Therefore, this method provides a feasible new approach for the treatment of severely displaced and unstable PHF in children.

## Data Availability

The datasets presented in this study can be found in online repositories. The names of the repository/repositories and accession number(s) can be found in the article/Supplementary Material.
